# Analysis of safety and efficacy of proton radiotherapy for optic nerve sheath meningioma

**DOI:** 10.1093/noajnl/vdae160

**Published:** 2024-09-21

**Authors:** Maximilian Y Deng, Sophie Rauh, Günes Anil, Jonathan W Lischalk, Laura Hahnemann, Tanja Eichkorn, Juliane Hörner-Rieber, Angela Paul, Elisabetta Sandrini, Philipp Hoegen-Sassmannshausen, Thomas Held, Sebastian Regnery, Lukas Bauer, Felix Sahm, Andreas von Deimling, Antje Wick, Wolfgang Wick, Christine Jungk, Sandro M Krieg, Klaus Herfarth, Jürgen Debus, Laila König

**Affiliations:** Translational Pediatric Radiation Oncology, Department of Radiation Oncology, Heidelberg University Hospital, Heidelberg University, Hopp Children’s Cancer Center (KiTZ), Heidelberg, Germany; National Center for Tumor Diseases (NCT), NCT Heidelberg, a Partnership Between DKFZ and Heidelberg University Hospital, Heidelberg, Germany; Heidelberg Institute for Radiation Oncology (HIRO) and National Center for Radiation Research in Oncology (NCRO), Heidelberg, Germany; Department of Radiation Oncology, Heidelberg University Hospital, Heidelberg University, Heidelberg, Germany; Heidelberg Ion-Beam Therapy Center (HIT), Department of Radiation Oncology, Heidelberg University Hospital, Heidelberg University, Heidelberg, Germany; National Center for Tumor Diseases (NCT), NCT Heidelberg, a Partnership Between DKFZ and Heidelberg University Hospital, Heidelberg, Germany; Heidelberg Institute for Radiation Oncology (HIRO) and National Center for Radiation Research in Oncology (NCRO), Heidelberg, Germany; Department of Radiation Oncology, Heidelberg University Hospital, Heidelberg University, Heidelberg, Germany; Heidelberg Ion-Beam Therapy Center (HIT), Department of Radiation Oncology, Heidelberg University Hospital, Heidelberg University, Heidelberg, Germany; National Center for Tumor Diseases (NCT), NCT Heidelberg, a Partnership Between DKFZ and Heidelberg University Hospital, Heidelberg, Germany; Heidelberg Institute for Radiation Oncology (HIRO) and National Center for Radiation Research in Oncology (NCRO), Heidelberg, Germany; Department of Radiation Oncology, Heidelberg University Hospital, Heidelberg University, Heidelberg, Germany; Department of Radiation Oncology, Perlmutter Cancer Center at New York University Langone Health at Long Island, New York, New York, USA; Heidelberg Ion-Beam Therapy Center (HIT), Department of Radiation Oncology, Heidelberg University Hospital, Heidelberg University, Heidelberg, Germany; National Center for Tumor Diseases (NCT), NCT Heidelberg, a Partnership Between DKFZ and Heidelberg University Hospital, Heidelberg, Germany; Heidelberg Institute for Radiation Oncology (HIRO) and National Center for Radiation Research in Oncology (NCRO), Heidelberg, Germany; Department of Radiation Oncology, Heidelberg University Hospital, Heidelberg University, Heidelberg, Germany; Heidelberg Ion-Beam Therapy Center (HIT), Department of Radiation Oncology, Heidelberg University Hospital, Heidelberg University, Heidelberg, Germany; National Center for Tumor Diseases (NCT), NCT Heidelberg, a Partnership Between DKFZ and Heidelberg University Hospital, Heidelberg, Germany; Heidelberg Institute for Radiation Oncology (HIRO) and National Center for Radiation Research in Oncology (NCRO), Heidelberg, Germany; Department of Radiation Oncology, Heidelberg University Hospital, Heidelberg University, Heidelberg, Germany; Clinical Cooperation Unit Radiation Oncology, German Cancer Research Center (DKFZ) Heidelberg, Heidelberg, Germany; Heidelberg Ion-Beam Therapy Center (HIT), Department of Radiation Oncology, Heidelberg University Hospital, Heidelberg University, Heidelberg, Germany; National Center for Tumor Diseases (NCT), NCT Heidelberg, a Partnership Between DKFZ and Heidelberg University Hospital, Heidelberg, Germany; Heidelberg Institute for Radiation Oncology (HIRO) and National Center for Radiation Research in Oncology (NCRO), Heidelberg, Germany; Department of Radiation Oncology, Heidelberg University Hospital, Heidelberg University, Heidelberg, Germany; Heidelberg Ion-Beam Therapy Center (HIT), Department of Radiation Oncology, Heidelberg University Hospital, Heidelberg University, Heidelberg, Germany; National Center for Tumor Diseases (NCT), NCT Heidelberg, a Partnership Between DKFZ and Heidelberg University Hospital, Heidelberg, Germany; Heidelberg Institute for Radiation Oncology (HIRO) and National Center for Radiation Research in Oncology (NCRO), Heidelberg, Germany; Department of Radiation Oncology, Heidelberg University Hospital, Heidelberg University, Heidelberg, Germany; Heidelberg Ion-Beam Therapy Center (HIT), Department of Radiation Oncology, Heidelberg University Hospital, Heidelberg University, Heidelberg, Germany; National Center for Tumor Diseases (NCT), NCT Heidelberg, a Partnership Between DKFZ and Heidelberg University Hospital, Heidelberg, Germany; Heidelberg Institute for Radiation Oncology (HIRO) and National Center for Radiation Research in Oncology (NCRO), Heidelberg, Germany; Department of Radiation Oncology, Heidelberg University Hospital, Heidelberg University, Heidelberg, Germany; Heidelberg Ion-Beam Therapy Center (HIT), Department of Radiation Oncology, Heidelberg University Hospital, Heidelberg University, Heidelberg, Germany; National Center for Tumor Diseases (NCT), NCT Heidelberg, a Partnership Between DKFZ and Heidelberg University Hospital, Heidelberg, Germany; Heidelberg Institute for Radiation Oncology (HIRO) and National Center for Radiation Research in Oncology (NCRO), Heidelberg, Germany; Department of Radiation Oncology, Heidelberg University Hospital, Heidelberg University, Heidelberg, Germany; Heidelberg Ion-Beam Therapy Center (HIT), Department of Radiation Oncology, Heidelberg University Hospital, Heidelberg University, Heidelberg, Germany; National Center for Tumor Diseases (NCT), NCT Heidelberg, a Partnership Between DKFZ and Heidelberg University Hospital, Heidelberg, Germany; Heidelberg Institute for Radiation Oncology (HIRO) and National Center for Radiation Research in Oncology (NCRO), Heidelberg, Germany; Department of Radiation Oncology, Heidelberg University Hospital, Heidelberg University, Heidelberg, Germany; Heidelberg Ion-Beam Therapy Center (HIT), Department of Radiation Oncology, Heidelberg University Hospital, Heidelberg University, Heidelberg, Germany; National Center for Tumor Diseases (NCT), NCT Heidelberg, a Partnership Between DKFZ and Heidelberg University Hospital, Heidelberg, Germany; Heidelberg Institute for Radiation Oncology (HIRO) and National Center for Radiation Research in Oncology (NCRO), Heidelberg, Germany; Department of Radiation Oncology, Heidelberg University Hospital, Heidelberg University, Heidelberg, Germany; Heidelberg Ion-Beam Therapy Center (HIT), Department of Radiation Oncology, Heidelberg University Hospital, Heidelberg University, Heidelberg, Germany; National Center for Tumor Diseases (NCT), NCT Heidelberg, a Partnership Between DKFZ and Heidelberg University Hospital, Heidelberg, Germany; Heidelberg Institute for Radiation Oncology (HIRO) and National Center for Radiation Research in Oncology (NCRO), Heidelberg, Germany; Department of Radiation Oncology, Heidelberg University Hospital, Heidelberg University, Heidelberg, Germany; Department of Neuropathology, Heidelberg University Hospital, Heidelberg University, and CCU Neuropathology, German Consortium for Translational Cancer Research (DKTK), German Cancer Research Center (DKFZ), Heidelberg, Germany; Department of Neuropathology, Heidelberg University Hospital, Heidelberg University, and CCU Neuropathology, German Consortium for Translational Cancer Research (DKTK), German Cancer Research Center (DKFZ), Heidelberg, Germany; Department of Neurology, Heidelberg University Hospital, Heidelberg University, Heidelberg, Germany; Clinical Cooperation Unit Neurooncology, German Consortium for Translational Cancer Research (DKTK), German Cancer Research Center (DKFZ), Heidelberg, Germany; Department of Neurology, Heidelberg University Hospital, Heidelberg University, Heidelberg, Germany; Department of Neurosurgery, University Hospital Heidelberg, Heidelberg University, Heidelberg, Germany; Department of Neurosurgery, University Hospital Heidelberg, Heidelberg University, Heidelberg, Germany; Heidelberg Ion-Beam Therapy Center (HIT), Department of Radiation Oncology, Heidelberg University Hospital, Heidelberg University, Heidelberg, Germany; National Center for Tumor Diseases (NCT), NCT Heidelberg, a Partnership Between DKFZ and Heidelberg University Hospital, Heidelberg, Germany; Heidelberg Institute for Radiation Oncology (HIRO) and National Center for Radiation Research in Oncology (NCRO), Heidelberg, Germany; Department of Radiation Oncology, Heidelberg University Hospital, Heidelberg University, Heidelberg, Germany; Department of Radiation Oncology, Perlmutter Cancer Center at New York University Langone Health at Long Island, New York, New York, USA; Heidelberg Ion-Beam Therapy Center (HIT), Department of Radiation Oncology, Heidelberg University Hospital, Heidelberg University, Heidelberg, Germany; National Center for Tumor Diseases (NCT), NCT Heidelberg, a Partnership Between DKFZ and Heidelberg University Hospital, Heidelberg, Germany; Heidelberg Institute for Radiation Oncology (HIRO) and National Center for Radiation Research in Oncology (NCRO), Heidelberg, Germany; Department of Radiation Oncology, Heidelberg University Hospital, Heidelberg University, Heidelberg, Germany; Heidelberg Ion-Beam Therapy Center (HIT), Department of Radiation Oncology, Heidelberg University Hospital, Heidelberg University, Heidelberg, Germany; National Center for Tumor Diseases (NCT), NCT Heidelberg, a Partnership Between DKFZ and Heidelberg University Hospital, Heidelberg, Germany; Heidelberg Institute for Radiation Oncology (HIRO) and National Center for Radiation Research in Oncology (NCRO), Heidelberg, Germany; Department of Radiation Oncology, Heidelberg University Hospital, Heidelberg University, Heidelberg, Germany

**Keywords:** optic nerve sheath meningioma, proton beam radiotherapy, visual outcome

## Abstract

**Background:**

Primary optic nerve sheath meningiomas (ONSMs) represent a group of benign tumors originating from the optic nerve sheath, typically causing painless, gradual onset monocular visual loss, which can result in blindness if left untreated. Radiation therapy represents an important treatment option for patients with ONSM, allowing for preservation and potential improvement in visual function. In particular, proton radiotherapy may enable a reduction of the side effects due to its physical advantage of an inverted dose profile with a steep dose gradient. The study investigates the visual acuity, local tumor control, and treatment-related toxicities following proton beam radiotherapy with a single institutional cohort comprising 32 patients treated for ONSM.

**Methods:**

Patients with primary ONSM, either histologically (16/32) or radiologically confirmed (16/32), which were treated at the Department of Radiation Oncology at the University Hospital Heidelberg (Germany) were assessed in regard to their visual outcomes, treatment toxicity, and local tumor control following radiotherapy according to response assessment in neuro-oncology criteria.

**Results:**

After a median follow-up time of 39.5 months, the 5-year local progression-free survival was estimated at 100%, with 84.4% of patients reporting improvement or stability in visual acuity during their last follow-up. Radiation-induced optic neuropathy (RION) was encountered in 9.4%.

**Conclusions:**

Our study demonstrates proton beam therapy as a safe and effective treatment alternative in the therapeutic management of ONSMs. RION represents a rare but dreaded complication after treatment. Future head-to-head comparisons with photon radiotherapy in a prospective setting are required to demonstrate a potential, additional clinical benefit.

**Key Points**

1. A stable or improved visual outcome can be reached in patients with optic nerve sheath meningiomas in up to 84% after proton beam radiotherapy.
2. Optic neuropathy represents a rare but dreaded complication in approximately 10%.
3. The dose-dependent risk of optic neuropathy may be higher—as presumed by TD5/5—in patients with optic nerve sheath meningiomas due to prior tumor-associated damages.

**Importance of the Study**

The gradual growth of primary optic nerve sheath meningiomas (ONSMs) may cause monocular visual loss, which can result in blindness if left untreated. Radiation therapy represents an important treatment option for patients with ONSM, allowing for preservation and potential improvement in visual function. In contrast, proton radiotherapy may enable a reduction of the side effects due to its physical advantage of an inverted dose profile with a steep dose gradient. The study summarizes our institutional experience in utilizing proton beam therapy in patients with ONSM, with 84.4% of patients reporting improvement or stability in visual acuity during their last follow-up and an estimated 5-year progression-free survival of 100%.

Primary optic nerve sheath meningiomas (ONSMs) represent a rare group of benign tumors originating from the optic nerve sheath, comprising about 1% to 2% of all meningiomas. Primary ONSM typically occurs in adult women in the fourth or fifth decades of life, with women being three times more likely to be affected than men.^1^ While primary ONSMs arise directly from the intraorbital dural sheath, a portion of intracranial meningiomas may extend anteriorly to involve the optic nerve. The tumor typically presents with a painless, gradual onset of monocular visual loss, which can—if left untreated—ultimately result in blindness.^2–4^ The diagnosis of ONSM can be confirmed radiologically using magnetic resonance imaging (MRI) and DOTATOC-PET.^5^ Surgical interventions are frequently omitted due to the significant risk of postoperative impact on visual function.^1,6^ The vascular supply of the optic nerve may be particularly at risk during surgery, which can result in severe visual impairment in up to 95% of patients.^4^ Radiation therapy (RT) represents an important treatment option for patients with ONSM, with the ability of preserving and improving visual function.^1,2,7,8^ While photon radiotherapy utilizes X-ray beams to irradiate a target volume, radiation dosage is unavoidably deposited in normal tissues beyond the target (eg, eyeball, retina, lens, and lachrymal gland). Proton beam RT (PBT) provides a physical dose advantage of an inverted dose profile with a steep dose gradient, enabling a reduction of the radiation dose for normal tissue, in particular for organs at risk.^9^ Recent results from a prospective study on WHO grade 1 meningioma following definitive or adjuvant PBT to a median dose of 50.4 GyRBE (range 48.6–61.2 GyRBE) has demonstrated a 5-year local progression rate of 6% and grade 3+ toxicity rate of only 2%.^10^ While prospective, randomized trials comparing PBT with conventional photon radiotherapy are still lacking for patients with meningiomas, PBT may be preferentially recommended in meningiomas with challenging tumor location (eg, skull-base meningiomas, optic-nerve sheath), where a dosimetric advantage to a critical organ-at-risk can reduce the risk of treatment-related toxicity.^11,12^

In 2002, Turbin et al. investigated a retrospective series of 64 patients—exclusively with ONSM—who were treated with observation, surgery alone, surgery combined with RT as well as RT alone. The cohort treated with RT alone, which received 40.0 to 55.0 Gy of conventional multiport or conformal external beam therapy, typically over 6 weeks, displayed the most favorable outcome regarding visual function.^13^ Furthermore, Arvold et al. have reviewed the outcome of 25 patients with ONSM treated with photon or proton radiation, or a combination of both. Stable disease was encountered in 95% of all patients during the last follow-up (median: 30 months; range: 3–168 months).^3^ An improvement or stability in visual acuity was achieved in 95%, while no severe treatment-related toxicities (CTCAE > grade 3) were found. Mild transient orbital pain or transient headache in 5% (*n* = 1), and asymptomatic retinopathy on MRI in 14% (*n* = 3) were reported in a subset of patients. There was no significant difference in visual acuity and treatment toxicities between proton beam and photon RT.^3^ Similar findings were reported by Moyal et al., comprising 15 patients with ONSM treated with proton beam radiotherapy (PBT).^14^ After a median follow-up of 22.4 months, local tumor control was observed in all patients, with an associated improvement or stability in visual acuity in 93.3% and an absence of treatment-related toxicities >grade 3.^14^ The low rate of eye-related treatment toxicities followed by proton beam RT (PBT) was previously illustrated in a meta-analysis comprising 41 studies, comparing six different RT techniques including 2-dimensional RT (2DRT), 3D-conformal RT (3DRT), stereotactic fractionated RT (SFRT), stereotactic radiosurgery (SRS), intensity-modulated RT (IMRT), and proton beam RT (PBT).^15^ Radiation-induced retinopathy was reported in 18% after 3DRT, 6.6% after IMRT and 2.2% after PBT.^15^

The present study reviews the visual acuity, local tumor control, and treatment toxicities following proton beam radiotherapy (PBT) in a single institutional cohort comprising 32 patients with ONSMs.

## Methods

### Patient Selection and Clinical Characteristics

Patients with primary ONSM, either histologically (16/32) or radiologically confirmed (16/32), which were treated at the Department of Radiation Oncology, University Hospital Heidelberg, Germany, between 2011 and 2022 were included in our study. Meningioma patients with secondary involvement of the optic nerve sheath were excluded from the study. Clinical patient characteristics were retrieved using the patient database of the Department of Radiation Oncology, University Hospital Heidelberg, Germany, and the National Cancer Registry. Informed consent was obtained for all patients and the study was approved by the Independent Ethics Committee of the Medical Faculty Heidelberg (S-293/2022).

### Radiotherapy Treatment Planning

**Figure 1.**
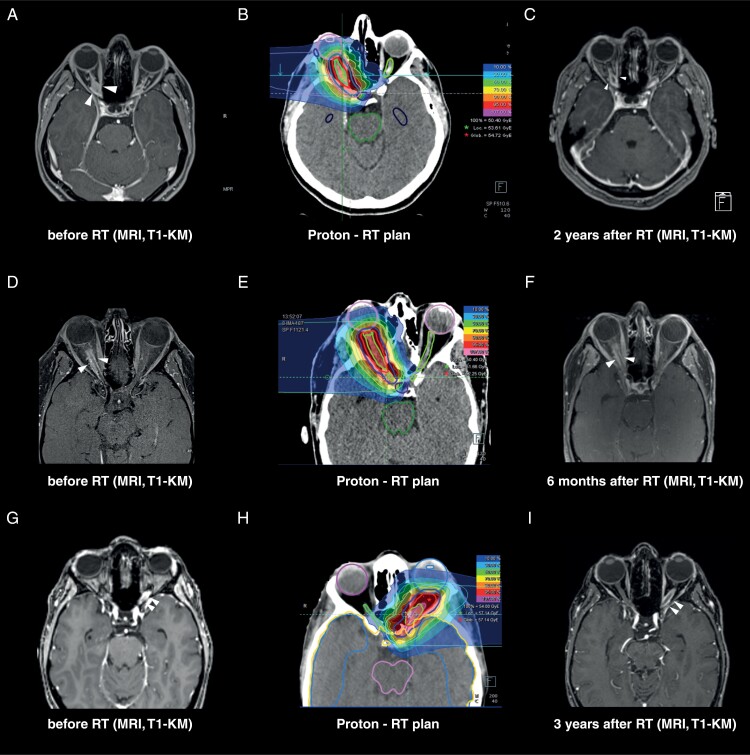
Treatment response following proton beam radiotherapy. Exemplary case with optic nerve sheath meningioma (ONSM)-03: (**A**) Right-sided ONSM (white arrows) on T1-weighted contrast-enhanced MRI before the start of proton radiotherapy. (**B**) Proton radiotherapy treatment plan with a total dose prescription of 50.4 Gy (RBE) in 1.8 Gy (RBE) single dose (100%), and colored isodose lines (in %). Proton radiotherapy was administered using a lateral beam arrangement to minimize radiation dose in the adjacent organs at risk (eg, lens, brain). In the specified beam direction, negligible radiation dose is deposited distal to the target volume or Bragg peak, eg, in the contralateral eye as preservation of contralateral visual outcome is crucial. (**C**) Increasing central T1-hypointensity within the ONSM as a sign of treatment response after 2 years following proton radiotherapy. Additional exemplary proton radiotherapy plans with corresponding MRI prior and following Radiation therapy (RT) (ONSM-04: **D-F**, ONSM-08: **G-I**).

Patients received highly conformal fractionated proton beam radiotherapy. Treatment planning was performed based on contrast-enhanced MR- and CT imaging for optimal target definition. Immobilization while receiving radiotherapy was accomplished using an individually manufactured head mask. Gross tumor volume (GTV) included the macroscopic tumor. The clinical target volume (CTV) comprised the GTV and a sub-clinical microscopic tumor region (eg, including the pre- or/and postoperative tumor bed, peritumoral edema if any, on CT/MRI at diagnosis), typically with a margin of 1–5 mm on the GTV. An isotropic margin of 3 mm was added to the CTV to define the planning target volume (PTV) for geometric uncertainties and physical inaccuracies of the beam. Organs at risk (eg, eyeball, retina, lens, lachrymal gland, chiasm) were previously manually contoured and dose constraints of normal tissue were followed according to Emami et al.^16^ and the QUANTEC data.^17^ Treatment plans were generated using Syngo PT-Planning (Siemens, Erlangen, Germany) and Raystation (Stockholm, Sweden). Proton dose was scaled with a constant relative biological effectiveness (RBE) factor of 1.1. Proton radiotherapy was administered using a lateral beam arrangement to minimize radiation dose in the adjacent organs at risk (eg, lens, brain). In the specified beam direction, the negligible radiation dose is imparted distal to the target volume or Bragg peak, eg, in the contralateral eye as preservation of the contralateral visual outcome is crucial (**Figure 1**). The Heidelberg Ion Therapy Beam Center (HIT) uses digital X-ray technology, which provides two orthogonal images of the skull, referenced to the treatment plan. The robotic treatment table allows a translation in 6 directions, including translational and rotational movements which facilitates an optimal positioning with less than 1mm positioning uncertainties possible.

### Assessment of Visual Outcomes, Treatment Toxicity, and Local Tumor Control Following Radiotherapy

Follow-up MRIs and clinical assessment were typically conducted 6–8 weeks after completion of RT and every 6–12 months thereafter. Visual acuity, visual field, and clinical signs (disc swelling, afferent pupillary defect, proptosis, or motility deficit) were assessed prior to radiotherapy and during clinical follow-up. Furthermore, the patient’s subjective perception of visual outcomes was evaluated during each visit. Visual deficits in the absence of tumor progression were scored as radiation-induced optic neuropathy (RION). Hormonal status was performed once a year to rule out radiation-induced hypopituitarism. Treatment toxicities were graded following the National Cancer Institute’s Common Terminology Criteria for Adverse Events (CTCAE) (version 5.0). A radiological response assessment was conducted according to the proposed criteria by the Response Assessment in Neuro-Oncology (RANO) Working Group.^18^

### Statistical Considerations

Progression-free survival (PFS) was determined from the start of radiotherapy until tumor progression and overall survival (OS) was calculated from the date of initial diagnosis to the last follow-up or death via Kaplan–Meier analysis. Cox proportional hazard models were used to identify prognostic factors. The influence on prognostic factors was assessed using the univariate Cox proportional regression model. Statistical analyses were conducted via Rstudio Version 4.0.2 (r-project.org). *P*-values < .05 were considered significant.

## Results

### Clinical Patient and Treatment Characteristics

In total, 32 patients with primary ONSM were included. The median age was 44.6 years (range: 13–64 years) at diagnosis. Female patients were more frequently encountered than males (male:female ratio: 3:1). Unilateral tumor manifestation was present in 91%, affecting the right eye in 34% (11/32) and the left eye in 56% (18/32) cases. Furthermore, 3 patients (9%) were affected by bilateral ONSM, resulting in bilateral irradiation. Growth patterns were characterized as tubular (16/32), fusiform (8/16), and exophytic (8/16) prior to treatment. Surgical (subtotal) resection for decompression was performed in 50% (16/32) prior to RT, while the other half received RT alone without upfront surgical resection. Exophytic meningiomas were mostly likely to receive prior surgery in 75% (6/8), as compared to ONSM with fusiform (50%, 4/8) or tubular presentation (37.5%, 6/16). Prior surgical intervention included biopsy (*n* = 1), tumor debulking (*n* = 10), decompression via optic canal opening (*n* = 4), and nerve sheath fenestration (*n* = 1; Table 1). All tumors, which underwent prior surgical resection (16/16) were diagnosed as WHO grade 1 meningiomas. Visual impairment was the predominant instigating factor to initiate RT, including a reduction in visual acuity (75%, 24/32), visual field deficits (59%, 19/32), and both exophthalmos and diplopia in 9% (3/32), respectively.

**Table 1.** Baseline and Treatment Characteristics

	Overall cohort (*n* = 32)	postoperative RT (*n* = 16)	definitive RT (*n* = 16)
Gender			
Female	24 (75%)	15 (94%)	9 (56%)
Male	8 (25%)	1 (6%)	7 (44%)
Age at primary diagnosis			
Median	44.6	42.3	46.1
Maximum-Minimum	13-64	14-62	13-64
Total dose in Gy (RBE)			
Median	54.0	54.0	54.0
Minimum–Maximum	50.4-56	50.4-56	50.4–54
Dose per fraction in Gy (RBE)			
1.8	30 (94%)	14 (87.5%)	16 (100%)
2.0	2 (6%)	2 (12.5%)	0 (0%)
PTV (ccm)	15.4	15.4 (range: 3.0–137.1)	14.6 (range: 2.9–69.9)
Tumor laterality			
Left	18 (56%)	10 (63%)	8 (50%)
Right	11 (34%)	5 (31%)	6 (38%)
Bilateral	3 (9%)	1 (6%)	2 (13%)
Symptoms before RT			
Vertigo	1 (3%)	0 (0%)	1 (6%)
Nausea	0 (0%)	0 (0%)	0 (0%)
Diplopia	3 (9%)	2 (13%)	1 (6%)
Exophthalmos	3 (9%)	0 (0%)	3 (19%)
Visual reduction	24 (75%)	11 (69%)	13 (81%)
Visual field defect	19 (59%)	10 (63%)	9 (56%)

The median time from diagnosis until RT was 2.2 years in the pre-operated cohort, compared to 1.0 years in treatment-naive ONSM patients. Overall, a median dose of 54.0 (RBE; range: 50.4–56.0 Gy; RBE) was administered in 5 fractions weekly with a median single dose of 1.8 Gy (RBE; range 1.8–2.0 Gy; RBE; **Table 1**).

### Treatment Toxicity and Visual Outcome

Detailed acute and chronic treatment toxicities are listed in **Table 2**. The most frequent symptoms comprised fatigue (56%, 18/32), focal radiogenic erythema (53%, 17/32), and intermittent cephalgia (25%, 8/32) during RT. The first follow-up (FU) visit was typically 8 weeks after completion of RT, where the radiogenic erythema vanished in all patients and fatigue improved (56% during RT vs. 22% in first FU). While most of the treatment-related symptoms generally improved following RT, xeropthalmia due to lacrimal dysfunction ≤grade 2 was reported in 9% of all patients during the first FU. No acute toxicities exceeding CTCAE grade 2 were encountered within 3 months after radiotherapy. Chronic treatment toxicities (after 3 months following completion of RT) were reported in a subset of patients, including xerophthalmia in 6% (2/32), intermittent cephalgia in 19% (6/32), and fatigue in 13% (3/32). One patient presented a transient asymptomatic hyperprolactinemia for 6 months, where no treatment was required.

**Table 2.** Treatment Toxicity (CTCAE ≤ 2°)

	Overall cohort (*n* = 32)	Resection (*n* = 16)	No Resection (*n* = 16)
Toxicities during RT			
Vertigo	3 (9%)	2 (13%)	1 (6%)
Nausea	4 (13%)	1 (6%)	3 (19%)
Diplopia	1 (3%)	1 (6%)	0 (0%)
Fatigue	18 (56%)	8 (50%)	10 (63%)
Cephalgia	8 (25%)	4 (25%)	4 (25%)
Radiogenic erythema	17 (53%)	9 (56%)	8 (50%)
Decrease in visual acuity	0 (0%)	0 (0%)	0 (0%)
Visual field loss	0 (0%)	0 (0%)	0 (0%)
First follow up (acute toxicities, <3 m)			
Vertigo	3 (9%)	1 (6%)	2 (13%)
Nausea	1 (3%)	1 (6%)	0 (0%)
Diplopia	0 (0%)	0 (0%)	0 (0%)
Fatigue	7 (22%)	3 (19%)	4 (25%)
Cephalgia	7 (22%)	4 (25%)	3 (19%)
Lacrimal dysfunction	3 (9%)	2 (13%)	1 (6%)
Decrease in visual acuity	0 (0%)	0 (0%)	0 (0%)
Visual field loss	0 (0%)	0 (0%)	0 (0%)
Chronic toxicities (>3 m)			
Vertigo	3 (9%)	2 (13%)	2 (13%)
Nausea	0 (0%)	0 (0%)	0 (0%)
Diplopia	0 (0%)	0 (0%)	0 (0%)
Fatigue	4 (13%)	1 (6%)	3 (19%)
Cephalgia	6 (19%)	3 (19%)	3 (19%)
Decrease in visual acuity	4 (13%)	2 (13%)	2 (13%)
Visual field loss	4 (13%)	3 (19%)	1 (6%)
RION	3 (9%)	2 (13%)	1 (6%)
Lacrimal dysfunction	2 (6%)	0 (0%)	2 (13%)

During the last follow-up, 37.5% (12/32) of all patients reported an improvement, or a stable visual status in 46.9% (15/32) following RT. Visual deterioration was reported in 15.6% (5/32): either due to tumor progression (2/32) or optic neuropathy (3/32; **Table 3**). All 3 patients were reported to exhibit a pale optic disc, indicative of optic neuropathy. These patients did not present any signs of retinopathy or tumor progression on MRI. Patient(-ONSM)-10 presented an initial improvement of the visual field after 3 months; however, gradual visual loss with a central scotoma was diagnosed after 11 months following RT. High-dose glucocorticoid treatment and 1 cycle of bevacizumab 7.5 mg/kg was administered subsequently, accomplishing a (then) stable visual status after a follow-up of 4 years. Patient(-ONSM)-17 presented a gradual occurrence of a central scotoma, with onset approximately 2 years after irradiation. High-dose glucocorticoid was administered but poorly tolerated. After discontinuation of the glucocorticoid treatment, the central scotoma remained unchanged during the last follow-up after 8 years. Patient(-ONSM)-19 presented a visual field loss after 9 months, which was treated with 6 cycles of bevacizumab 10 mg/kg biweekly, achieving a stable visual status thereafter. Both patients presenting RION in the absence of tumor progression had preexisting conditions prior to treatment: ANA-positive connective tissue disease and a smoking history of 15–20 pack years (ONSM-17), and cutis marmorata telangiectatica congenita and factor V Leiden with reported events of sinus venous thrombosis (ONSM-19). Additional information on comorbidities, including the presence of vascular disease and coronary heart disease, hypertension, diabetes, obesity, hypercholesterolemia, thyroid disease, and smoking history is in Supplementary Table 1. No significant difference in visual outcome was observed between the pre-operated and treatment-naive naive ONSM patients following RT. Median PTV was 15.43 mL (range: 2.86–137.11 mL) for the entire cohort and 14.60 mL (range: 2.99–88.43 mL) for the non-operated subgroup. However, there was no association between the PTV volume, dose maximum at the optical system, and the visual outcome (**Supplementary Figure 1**). Additional dosimetric information related to the organs at risk (eg, optic nerve, optic chiasm, eye, and lens) are listed in Supplementary Table 1.

**Table 3.** Visual Improvement and Deterioration During Last Follow-up

Patient-ID	Prior surgical intervention	Growth pattern	Total dose in Gy	PTV (ml)	Dmax (optical system) in Gy	Visual improvement	Comment
ONSM-01	optic canal opening	tubular	50.4	8.67	51.80	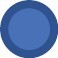	
ONSM-02	No	tubular	52.2	4.5	52.92	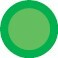	Visual field improved
ONSM-03	No	tubular	50.4	6.28	54.02	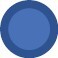	
ONSM-04	No	tubular	50.4	9.98	52.25	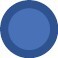	
ONSM-05	No	tubular	50.4	2.99	51.56	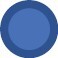	
ONSM-06	optic canal opening	tubular	54.0	17.71	51.85	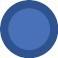	
ONSM-07	No	fusiform	52.2	20.59	53.66	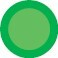	Δ 0.25cc
ONSM-08	No	tubular	54	9.99	56.47	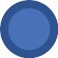	
ONSM-09	No	exophytic	50.4	17.1	51.57	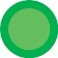	Δ 0.25cc
ONSM-10	debulking	exophytic	52.2	16.57	51.99	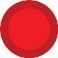	**RION**, Δ 0.95cc
ONSM-11	No	fusiform	52.2	16.66	53.47	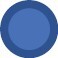	
ONSM-12	debulking	fusiform	54.0	9.21	50.64	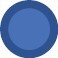	
ONSM-13	optic canal opening	exophytic	52.2	4.88	52.97	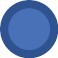	
ONSM-14	debulking	exophytic	54.0	137.11	56.83	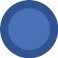	
ONSM-15	optic canal opening	exophytic	54.0	7.22	48.35	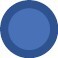	
ONSM-16	fenestration	exophytic	52.2	2.86	52.85	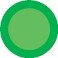	Visual field improved
ONSM-17	No	fusiform	52.2	22.03	54.84	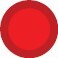	**RION**, Δ 0.75cc
ONSM-18	debulking	tubular	52.2	21.04	54.59	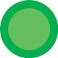	Δ 0.50cc
ONSM-19	biopsy	tubular	52.2	69.46	53.52	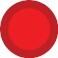	**RION**, Δ 0.60cc
ONSM-20	debulking	fusiform	52.2	14.29	53.93	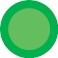	Δ 0.40cc
ONSM-21	No	tubular	52.2	18.16	54.09	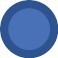	
ONSM-22	No	tubular	54.0	88.43	51.58	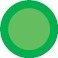	Δ 0.20cc
ONSM-23	debulking	exophytic	54.0	103.11	52.1	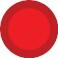	**Progressive Disease**, visual field
ONSM-24	No	tubular	50.4	22.41	53.11	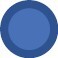	
ONSM-25	No	fusiform	52.2	12.54	53.69	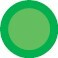	Visual field improved
ONSM-26	No	exophytic	52.2	69.92	53.67	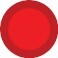	**Progressive Disease**, Δ 1.00cc
ONSM-27	debulking	fusiform	54.0	31.02	56.24	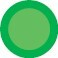	Δ 0.50cc
ONSM-28	debulking	tubular	56.0	48.46	59.81	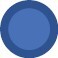	
ONSM-29	No	tubular	54.0	7.03	54.98	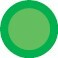	Visual field improved
ONSM-30	debulking	tubular	54.0	6.38	57.57	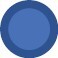	
ONSM-31	debulking	fusiform	52.2	11.18	52.57	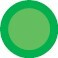	Δ 0.60cc
ONSM-32	No	tubular	50.4	4.27	51.25	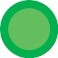	Δ 0.10cc

Visual status indicated in (1) blue = stable, (2) green = improvement and (3) red = deterioration, as compared to the baseline prior to proton beam therapy. Prior surgical intervention included biopsy (*n* = 1), debulking (*n* = 10), and decompression via optic canal opening (*n* = 4) and nerve sheath fenestration (*n* = 1). Visual acuity testing was conducted **with correction (cc).**

### Survival and Local Control

The median follow-up was 39.5 months (range: 11–128 months), with an estimated 5-year PFS of 100%. While a partial response, defined as ≥50% decrease in area relative to baseline by the RANO working group was not observed after RT, 3/32 meningiomas demonstrated an increasingly central T1-hypointensity, which may be interpreted as treatment response with a decrease in cellularity (**Figure 1**).^18^ After a period of >5 years, 2 patients demonstrated tumor progression after 66 and 99 months, respectively. Both patients who went on to experience progression did not exhibit a central T1-hypointensity following RT.

## Discussion

The management of optic nerve sheath meningioma (ONSM) is a challenging interdisciplinary task due to its close vicinity to critical structures including the optic system and adjacent organs at risk (eg, lacrimal gland, pituitary gland). Radiotherapy for ONSM was reported to represent a favorable therapeutic approach, particularly in patients not safely amenable to surgical resection.^1,6^ In general, proton beam therapy holds certain innate physical and biological advantages (eg, Bragg peak) over conventional photon radiotherapy by generating a steep dose gradient with rapid fall-off at the distal end. Particularly in the specified beam direction, the negligible radiation dose is deposited distally to the target volume or Bragg peak, eg, in the contralateral eye as preservation of the contralateral visual outcome is crucial. Consequently, proton beam therapy may facilitate an improved dose sparing in the adjacent critical anatomic structures on a dosimetric level—however, long-term clinical follow-up is required to substantiate these amenities. The current study presents our institutional experience of applying proton beam therapy in patients with ONSM in both adjuvant and definitive settings.

With a median follow-up of 39.5 months, the 5-year local PFS was estimated at 100%, with 84.4% of patients reporting an improvement or at least stability in visual acuity during their last follow-up. An extended clinical follow-up is warranted due to the anticipated delayed tumor progression during the course of the disease, particularly considering the generally benign nature of WHO grade 1 meningiomas. Our findings are in accordance with previous reports on local tumor control in grade 1, or non-resected meningiomas following radiotherapy with a local 5-year PFS >90%. However, no final conclusion can be drawn as to the long-term local tumor control, yet, due to the short follow-up period for grade 1 meningiomas in our series.^5,19–22^ The vast majority of side effects were transient and self-limiting, including intermittent cephalgia, fatigue, and mild skin erythema. Acute toxicities exceeding CTCAE grade 2 were not observed in our cohort. However, RION was encountered in 9.4% (3/32) which is thought to represent a rare but dreaded complication. The visual outcome in our study cohort is in accordance with previous reports: Hage et al. published data on 60 patients with primary ONSM treated with either definitive PBT (51.7%, *n* = 31) or adjuvant PBT (48.3%, *n* = 29). Among all patients, 15% suffered from RION/retinopathy, leading to rapid progressive visual loss.^23^ The study revealed no significant tumor growth and maintained visual acuity after a median follow-up of 48 months. The authors suggested that surgery prior to PBT did not additionally impact visual acuity. Treatment toxicities included mild dermatitis, alopecia, pain, and asthenia; however, no further details were provided.^23^ Furthermore, a meta-analysis by Vaishnav et al. has reported that nearly 90% of patients noted either stability or improvement of visual acuity following RT, with a similar local control rate of 99.8%.^24^ Our previous analysis of photon radiotherapy for ONSM has shown that prior surgical resection is a negative prognostic factor for visual outcome.^2^ However, our study on proton radiotherapy did not demonstrate a difference in visual outcome between pre-operated and non-operated patients, which may be attributed to the small cohort sizes. Typical signs of retinopathy (eg, retinal hemorrhage) were absent in our cohort. However, it should be noted that previous studies, eg, Hage et al. categorized patients (5/60) with patchy peripapillary or interpapillomacular ischemia (ie, between the optic nerve head and the center of the macula) as radiation-induced retinopathy.^23^ While ischemia in these areas is often associated with (anterior) ischemic optic neuropathy, we have classified these events as RION.

Detailed information on dosimetry was available in our study cohort. Two patients presented optic neuropathy although the maximal dose (Dmax) did not exceed 54 Gy as TD_5/5_ at the optical system, a reported threshold of an estimated 5% risk of severe complications within 5 years after irradiation.^16,17^ Long-term tumor-dependent pressure damage, or prior surgical intervention, may result in a disruption of the vasa nervorum et vasorum of the optic nerve which may not be apparent based on MRI. Further, additional patient-related factors and comorbidities may attribute to an increased risk of optic neuropathy following RT. In fact, presumably relevant preexisting conditions were reported in 2/3 of patients presenting optic neuropathy in the absence of tumor progression (eg, ANA-positive connective tissue disease, history of smoking, factor V Leiden, reported events of sinus venous thrombosis). Thus, given that the resulting optic neuropathy may be multifactorial, the risk estimation of radiation-attributed side effects as presumed by TD_5/5_ remains particularly challenging.^25–27^ Previous case reports have illustrated a potential benefit of bevacizumab in patients with radiation optic neuropathy.^28^ In our series, two patients with optic neuropathy were treated with bevacizumab with 6 cycles in 10 mg/kg and 1 cycle in 7.5 mg/kg, respectively. Both patients presented a long-term stable visual outcome following bevacizumab administration—however, clinical evidence for bevacizumab in the treatment of radiation-associated optic neuropathy remains largely anecdotal. Overall, a low rate of xerophthalmia through lacrimal dysfunction (9%, D_median_ = 14, 64 GyRBE) or chronic hypopituitarism (D_median_ = 12, 11 GyRBE) was encountered in our study cohort, which may be attributed to the favorable dosimetry through proton beam therapy by reducing radiation dose in the adjacent organs at risk, particularly in the specified beam direction distal to the target volume or Bragg peak (**Figure 1**).

This study should be interpreted in the context of its limitations: clinical follow-up data were obtained retrospectively, and therefore susceptible to biases inherent to retrospective studies. Treatment decisions were largely driven by providers and patients, potentially leading to differences in baseline clinical and molecular characteristics. There was no difference apparent in regard to the treatment volume (PTV) between treatment-naïve and resected patients—however, it may be anticipated that the extent of the initial tumor prior to neurosurgical decompression was larger (vs. non-resected patients), making it difficult to directly compare both cohorts in terms of treatment toxicities as initial symptoms may vary. Consequently, no conclusions can be drawn as to whether radiotherapy alone, surgery alone, or surgery with adjuvant RT may be recommended for various tumor extent, as our study did not include patients after surgery alone. Furthermore, sufficient tumor material for molecular testing was not available, resulting in a lack of molecular profiling and risk stratification (eg, DNA methylation profiling, gene-expression marker), which would have been beneficial for the interpretation of clinical outcome data or response prediction.^29–33^

Overall, our study suggests that proton bean therapy represents a safe and effective treatment option in patients with optic nerve sheath meningioma, with a stable or improved visual performance in 84.4% after a median follow-up of >4 years.

## Supplementary Material

vdae160_suppl_Supplementary_Table

vdae160_suppl_Supplementary_Table_S1_Figure_S1
