# Supplementary material for: Analysis of safety and efficacy of proton radiotherapy for optic nerve sheath meningioma
Source: Neurooncol Adv. 2024 Sep 21;6(1):vdae160. doi: 10.1093/noajnl/vdae160 (PMC11491494; doi:10.1093/noajnl/vdae160)
Supplement: vdae160_suppl_Supplementary_Table_S1_Figure_S1 [file vdae160_suppl_supplementary_table_s1_figure_s1.docx]

**Supplementary Figure and Tables**

**Suppl. Figure 1. Potential influence of clinical and dosimetric parameters on visual deterioration.**

Abbreviations: OAR_Dmax maximum dose in the optical system, PTV planning target volume. HR hazard ratio, CI 95% confidence interval.


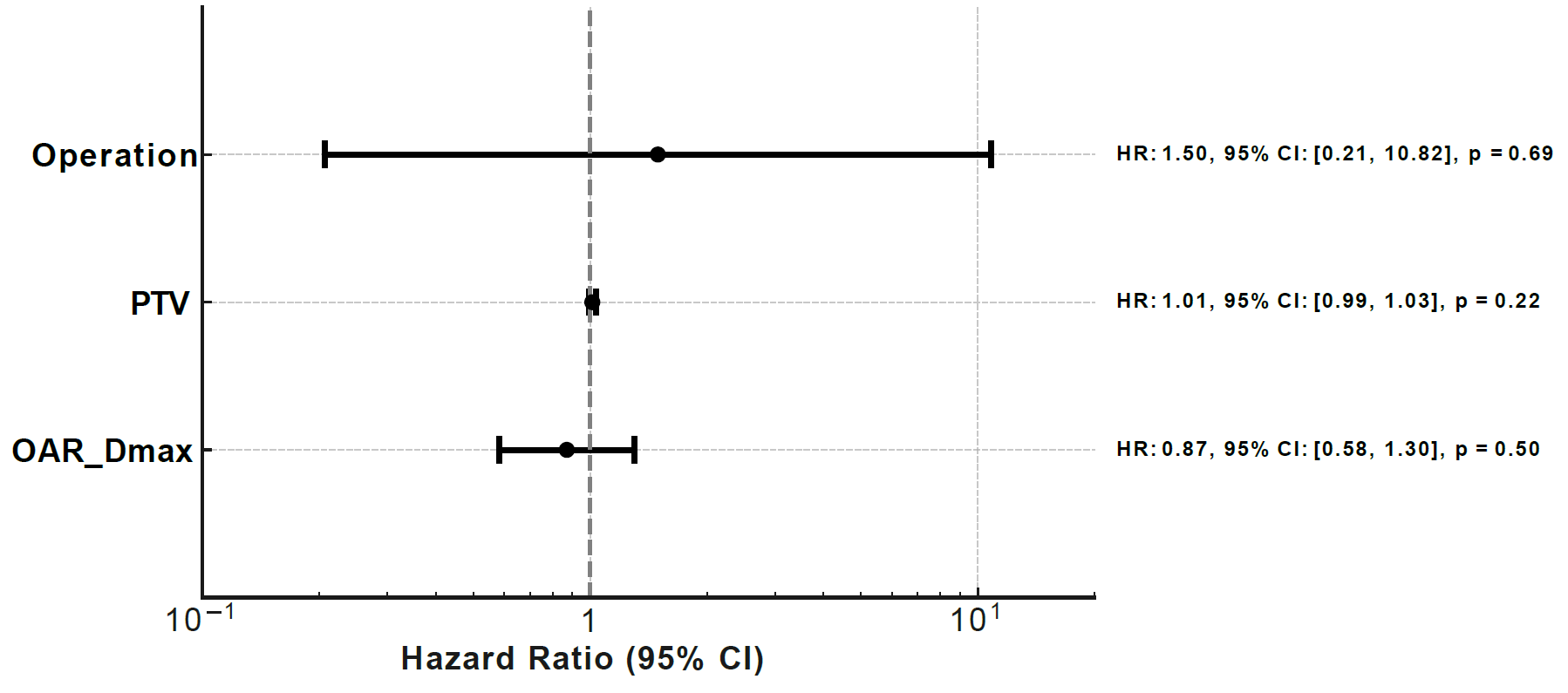


**Suppl. Table 1. Overview of dose parameters and comorbidities**

See Excel-Table.

**Suppl. Table 1. Overview of dose parameters and comorbidities**

**Summary of the literature**

| **Publication** | **n** | **Median age at RT** | **RT modality** | **Median total dose** | **Previous surgery** | **Median follow-up since RT** | **Local control at last follow-up:** | **Visual acuity at last follow-up** | **Toxicity** | **Optic neuro-pathy** |
| --- | --- | --- | --- | --- | --- | --- | --- | --- | --- | --- |
| Adeberg S et al. 2011 (2) | 40 | 44 years (range: 17-83 years) | FSRT (Photon)  Adjuvant: 21  Definitive: 19 | 54 Gy (range 25-66 Gy) | No further details | 60 months (range 4-228 months) | 100% | Total: 95% (38/40)  Improvement: 44.4% (12/27 patients with pre-existing impairment) | Mild acute toxicity | None of the patients developed neuropathy, retinopathy or brain necrosis |
| Arvold ND et al. 2009 (3) | 25 | 49 years (range: 9-87 years) | FCRT  Photon: 13/25  Proton: 9/25  bimodal: 3/25 | 50.4 Gy RBE (range, 45-59.4 Gy RBE) | None: 68% (17/25)  Biopsy: 20% (5/25)  Subtotal resection: 12% (3/25) | 30 months (range: 3-168 months) | 95% | 95% (21/22)  Photon: 100% (11/11)  Proton: 87.5% (7/8)  Bimodal: 100% (3/3) | CTCAE < 3 | Asymptomatic retinopathy:  3/22  Improved/ stable vision: 2/22 |
| Metellus et al. 2011 (8) | 9 | 47 years (range: 39– 59 years) | FCRT  Adjuvant RT: 22.2% (2/9)  Definitive RT: 77.8% (7/9) | 50.4 Gy (range: 36-54 Gy) | partial resection: 100% (2/2) | 90 months (range: 61–151 months) | 100% | 100%  Improvement: 77.8% (7/9)  Stability: 22.2% (2/9) | Eyelid edema: 11% (1/9) | Retinopathy: 11% (1/9) |
| Turbin RE et al. 2002 (13) | 59  (originally 64 patients, 5 excluded from statistical analysis) | 47.1 years (range: 17–81 years) | multiport or conformal external beam therapy (Photon)  Observation only: 13  Surgery only: 12  Adjuvant RT: 16  Definitive RT: 18 | 40.0-55.0 Gy | Surgery only: 12 (biopsy/STR = 4, GTR=8)  adjuvant RT: 16 | 150.2 months (range: 51–516 months) | Total: 67.2% (43/64) | Observation only/surgery only/adjuvant RT: significant decrease in visual acuity  definitive RT: most favorable outcome regarding visual function | Surgery only: 66.7% (8/12)  Adjuvant RT: 62.5% (10/16)  Definitive RT: 33.3% (6/18) | Retinopathy or vascular occlusion:  Adjuvant RT: 12.5% (2/16)  definitive RT: 22.2% (4/18) |
| Moyal L et al. 2014 (14) | 15 | 41.8 years | PBT as primary treatment: 6/15  adjuvant:  5/15  following observation: 4/15 | 52.2 Gy RBE | Previous surgery: 33.3% (5/15) | 22.4 months (8-79 months) | 100% | 93.3% (14/15) | CTCAE < 3 | Not documented |
| de Melo LP et al. 2021 (15) | meta-analysis: 41 studies, 736 ONSM | / | 6 different modalities (2DRT, 3DRT, FSRT, IMRT, PBT, SRS) | 2DRT: 55 Gy (46–55 Gy)  3D-RT: 53 Gy (50–58 Gy)  FSRT: 52 Gy (43– 54 Gy)  IMRT: 50 Gy (44–50 Gy)  PBT 52.2 Gy RBE  SRS: 13.3 - 17.0 Gy | / | 47 months (20–144 months) | Total: 97,4%  2DRT: 90% (78–100%)  3CRT: 96.1% (91–100%)  FSRT: 97.8% (96–99%)  IMRT: 97.1% (93–100%)  PBT: 96.3% (89–100%)  SRS: 90.6% (96–98%) | Improvement: 45% (0–100%)  Stability: 40% (0–100%)  Deterioration: 15% (0–50%) | Complications from 18 studies:  Cataract: 18%  dry eye: 11%  pituitary disfunction: 9%  optic neuritis: 6,6%  orbital pain: 4.4%  iritis: 4.4% | Radiation-induced retinopathy:  3DRT: 18%  FSRT: 18%  IMRT: 6.6%  PRT: 2.2% |
| Hage R et al. 2021 (23) | 60 | 45.2 years (34.1–56.3 years) | Proton beam RT (PBT)  Definitive PBT: 51.7% (31/60)  Surgery + PBT: 48.3% (29/60) | 52.2 Gy RBE (range: 49.3 – 54 Gy RBE) | Biopsy: 6  Biopsy + Resection: 6  Intracranial extension resection: 15  Other surgeries: 3 | 48 months (IQR=24–63 months)  (5 Patients lost to follow-up) | 100% | Visual acuity: no significant changes post-PBT  and  no significant difference between definitive RT and RT + prior surgery  Visual field: Improvement or stability:  85.45% (47/55) | moderate epithelitis, infrequent limited slight alopecia, pain, and asthenia (no further details) | RION: radiation optic neuropathy: 1.6%  RR: radiation retinopathy:  13% (7/60)  (surgery + PBT: 17%, definitive PBT: 6%) |
| Vaishnav YJ, Singh R et al. 2022 (24) | meta-analysis: 20 studies, 444 patients | 46.75 years (range: 9-87 years) | definitive, adjuvant, or salvage radiation | 50.4 Gy (range: 50-58 Gy) | / | 51.15 months (range, 1-248.4 months) | 99.8%  (Information on LC in 19 studies with 412 patients) | 89.7%  (information on visual acuity in 19 studies with 379 patients) | xeropthalmia: 10.1% | radiation-induced retinopathy/ neuropathy: 7.2% |
